# How Gut Microbiota Influence Healthy Aging: Overview of Reviews

**DOI:** 10.3390/geriatrics11040107

**Published:** 2026-08-17

**Authors:** Tejas Ganesh Todmal, Rabia Bibi, Giovanni Cangelosi, Massimiliano Panella, Alice Masini

**Affiliations:** 1 Department of Health Sciences, University of Eastern Piedmont, Via Solaroli 17, 28100 Novara, NO, Italy; 20054811@studenti.uniupo.it; 2 Department of Translational Medicine, University of Eastern Piedmont, Via Solaroli 17, 28100 Novara, NO, Italy; massimiliano.panella@med.uniupo.it (M.P.); alice.masini@uniupo.it (A.M.); 3 School of Pharmacy, Experimental Medicine and “Stefani Scuri” Public Health Department, University of Camerino, 62032 Camerino, MC, Italy; giovanni01.cangelosi@unicam.it; 4 Direzione Medica dei Presidi Ospedalieri, Azienda Ospedaliero-Universitaria di Alessandria, 15121 Alessandria, AL, Italy

**Keywords:** microbiota, longevity, diet, Mediterranean diet, dysbiosis, overview of reviews

## Abstract

**Background**: Gut microbiota plays a key role in the aging process, with age-related microbial shifts contributing to chronic inflammation, metabolic dysfunction, frailty, and cognitive decline. Despite growing interest, an integrated synthesis of how diet and lifestyle modifications influence gut microbiota remains limited. This overview of reviews summarizes the current evidence on how dietary and lifestyle interventions shape microbial composition and affect aging outcomes. **Methods**: Following the PRISMA guidelines, we searched PubMed and Scopus for English-language reviews (January 2023–October 2025) including human adults aged ≥ 18 years, evaluating dietary or lifestyle interventions, and reporting gut microbiota with healthy-aging outcomes. Quality of the reviews was appraised using the SANRA tool. **Results**: Mediterranean and plant-based diets, calorie restriction, and microbiota-targeted approaches such as probiotics, prebiotics, symbiotics, and fecal microbiota transplantation were associated with increases in short-chain fatty acid-producing bacteria, including *Faecalibacterium*, *Bifidobacterium*, *Lactobacillus*, and *Akkermansia muciniphila*, while reducing pro-inflammatory taxa. These changes were linked to improved metabolic and immune function, reduced inflammaging, lower frailty, and greater physical resilience. Cognitive benefits included decreased neuroinflammation and a lower risk of Alzheimer’s and Parkinson’s diseases. **Conclusions**: Maintaining microbial balance through targeted dietary strategies may support healthier, more resilient aging, offering practical insights for public health and clinical nutrition planning.

## 1. Introduction

The gut microbiota is a complex collective system of bacteria, viruses, archaea, and fungi that regulates critical physiological functions ranging from digestion and immune defense to metabolism and neural connectivity via the gut–brain axis [1]. The current evidence depicts that the gut microbiota plays a fundamental role in the trajectory of the aging process [2], a relationship that has grown increasingly relevant as the global aging population expands rapidly; according to the OECD (Organization for Economic Co-operation and Development), in the 2025 report, the proportion of individuals aged 65 years and older has increased substantially between 1960 and 2022 [3]. Interestingly, this paradigm shift in the older age population has pushed forward certain healthcare projects promoting “healthy aging,” defined as the maintenance of functional ability and sustained well-being [4,5].

As individuals advance in age, the composition of this microbial community undergoes significant alteration, characterized by a decrease in beneficial short-chain fatty acid (SCFA)-producing bacteria, namely, *Bifidobacterium* and *Faecalibacterium prausnitzii*, alongside a concurrent increase in pro-inflammatory species [6]. This microbial imbalance contributes directly to chronic, low-grade systemic inflammation (also known as inflammaging), metabolic dysfunction, and cognitive decline, thereby accelerating frailty and functional impairment in older adults [7]. The stability of this system is critical yet often compromised; in fact, more than 40% of the global population suffers from some sort of gastrointestinal disorder, creating a burden that disrupts quality of life and increases healthcare utilization [7]. Because dietary and lifestyle interventions can modulate gut microbial composition and, in turn, this inflammatory pathway, they represent a plausible strategy for mitigating both the physical and cognitive consequences of age-related dysbiosis [1,7]. In contrast, balanced and diverse gut microbiota lead to improved immunity, metabolic regulation, and neuroprotection, all of which contribute to healthy aging. Recent data have identified microbial signatures correlated with biological age, with genera such as *Prevotella* linked to accelerated aging, while others, including *Akkermansia* and *Alistipes*, are associated with healthier aging trajectories [3,8]. Mendelian randomization analyses further suggest a potential causal role of the microbiome in driving metabolic decline, frailty, and neuroinflammation in older adults [9].

Given this relationship, several interventions have been proposed, with prebiotics showing the strongest clinical evidence for improving mobility, reducing inflammation, and enhancing microbial balance in elderly individuals [10]. Among dietary approaches is the Mediterranean Diet (MD), derived from the countries surrounding the Mediterranean Sea. This diet mainly emphasizes the intake of a plant-based diet and low intake of meat products and has also demonstrated well-established clinical and metabolic benefits across chronic diseases in general [11,12]. MD is also rich in fibers, polyphenols, and plant-based foods that support essential microbes such as *Bifidobacterium*, *Faecalibacterium prausnitzii*, and *Roseburia*, which produce SCFAs beneficial for intestinal and systemic health, a mechanism that helps explain its capacity to enhance SCFA-producing taxa [13]. Large intervention studies, including the NU-AGE trial, show that adherence to the MD in older adults increases gut microbial diversity and abundance of health-associated taxa while reducing markers of frailty and systemic inflammation, with concomitant improvements in cognitive performance and mobility [14]. These effects are mediated in part by increased SCFA production and decreased pro-inflammatory metabolites, such as secondary bile acids and trimethylamine-N-oxide (TMAO). Conversely, Western diets include processed food, refined sugars, and saturated fats, which lead to microbial imbalance and chronic low-grade inflammation, which may accelerate the aging process [15].

Although several recent reviews have examined gut health, diet, and longevity, an integrated synthesis exploring the interrelationships between gut health, dietary patterns, and aging remains limited. Therefore, we conducted an overview of reviews to comprehensively examine the interconnected roles of gut health in the aging process. The aim of this overview of reviews is to summarize the evidence on the relation between how dietary and lifestyle interventions affect gut microbiota composition and function, as well as the effect of microbial changes on healthy aging. Moreover, this overview distinctly enhances the gap in research by integrating updated and current evidence that can provide a comprehensive and detailed view of the current knowledge to support not only longevity but also overall quality of life in older adults.

## 2. Materials and Methods

### 2.1. Study Selection and Screening

The present work is an overview of reviews performed systematically in accordance with the Preferred Reporting Items for Systematic Reviews and Meta-Analyses (PRISMA) guidelines to ensure transparency and replicability [16]. The completed PRISMA 2020 checklist is provided in the Supplementary Materials (Table S1). All the records from the databases are exported in Microsoft Excel (Microsoft 365; Microsoft Corporation, Redmond, WA, USA) spreadsheets, and duplicate articles were removed. Two independent reviewers (R.B and T.G.T) screened the remaining titles and abstracts. Subsequently, the full texts of all potentially relevant articles were screened and assessed for final inclusion, and only those reviews meeting all specified inclusion criteria were ultimately included in the final synthesis.

### 2.2. Search Strategy

The search was conducted across two major scientific databases: PubMed and Scopus. The search was strictly limited to articles published in English with full-text availability and a publication date between 1 January 2023 and 31 October 2025. This timeframe was selected to capture the most recent narrative and mini-review literature on gut microbiota and healthy aging, reflecting the rapid expansion of research in this field to provide a contemporary synthesis of the latest literature. The search strategy included several MeSH keywords combined and/or separated by the Boolean operators, as provided in Supplementary Materials Table S2.

### 2.3. Eligibility Criteria

The review followed a structured process using the PICOS guideline [17], adapted for an Overview of Reviews, to guide the selection of relevant studies.

1. P (Population): Human adults aged ≥ 18 years, all sexes.
2. I (Intervention): Dietary (e.g., Mediterranean, plant-based diets, fiber/polyphenol intake, or other modifications).
3. C (Comparison): Standard diets or alternative patterns (where applicable).
4. O (Outcome): Healthy longevity, including changes in gut microbiota diversity, SCFA production, functional metabolites, and physical, cognitive, microbial, and overall health.
5. S (Study design): Review studies.

### 2.4. Data Extraction and Quality Assessment

We extracted the data in a standardized Microsoft Excel spreadsheet. Extracted data included the review characteristics, such as sample characteristics (such as the total number of participants, age, and sex presented as totals and percentages where available), author’s name, publication year, country, type of review, and objectives. Finally, the findings from each review were summarized in the tables and text, such as specific intervention focus (e.g., dietary patterns or microbiota-related interventions). Two independent researchers (R.B. and T.G.T) assessed the quality of included studies using the Scale for the Assessment of Narrative Review Articles (SANRA) [18]. The scale is a six-item critical appraisal tool specifically developed for narrative reviews. Each item is scored on an integer scale from 0 (low quality), 1 (medium quality), and 2 (high quality), yielding a maximum possible sum score of 12. We use this sum score to quantify the overall quality of each narrative review, where higher scores indicate better methodological and reporting quality.

## 3. Results

### 3.1. Identification of Studies

We identified 849 records from the databases, while no relevant records were identified from the external sources. After removing 19 duplicates, 830 articles underwent title and abstract screening, leading to the exclusion of 777 studies due to irrelevance, inadequate data, or lack of focus on gut microbiota and lifestyle factors.

**Figure 1:**
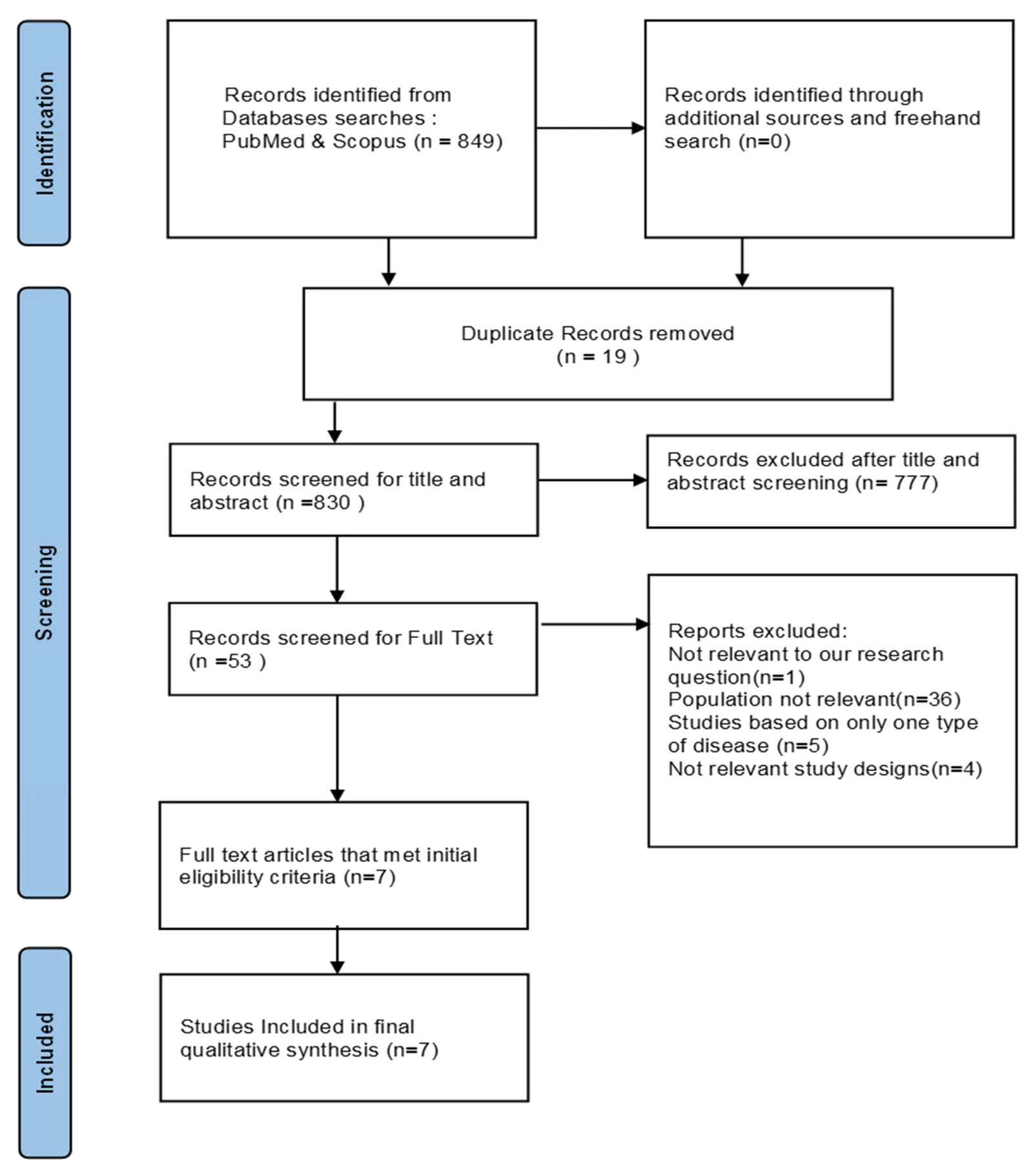
PRISMA flow chart of the included studies.

Subsequently, 53 full-text articles were reviewed for eligibility. Ultimately, seven studies met all inclusion criteria and were incorporated into the qualitative synthesis, providing key insights into the relationship between gut microbiota, diet, and lifestyle in healthy aging. Finally, we included seven articles that were included for our qualitative synthesis. The entire process is summarized in Figure 1 following the PRISMA guidelines.

### 3.2. General Characteristics of Included Studies

The general characteristics of the included studies are summarized in Table 1. The included studies, published between 2023 and 2025, originated from a diverse set of countries, including one from Greece, three from China, one from the USA, Venezuela, and Japan. From these seven studies, six are narrative reviews, and one is a mini-review [8,17,18,19,20,21,22]. Sample characteristics varied substantially in terms of population, sex, and age ranges, while most reviews did not report participant demographics. Gyriki et al. [8] included data from 2841 adults to centenarians of mixed sex, and Xu X et al. [20] focused on 110 middle-aged and older women. However, across all reviews, the main theme was the exploration of how gut microbiota composition shifts with aging and how such changes influence longevity, immune function, metabolic health, and age-related diseases.

All the included studies also investigated comparable intervention strategies to improve microbiota composition and promote healthy aging. Reviews by Xu X et al. [17], Upadhyay [20], and Salazar et al. [21] all discussed the use of probiotics, prebiotics, dietary modulation, and fecal microbiota transplantation (FMT) as potential therapeutic approaches. Similarly, Gyriki et al. [8] and Li et al. [19] evaluated microbiota-targeted interventions positioned as tools to enhance lifespan and reduce age-related disease risk. A unique yet related perspective was provided by Shintani et al. [25], who examined calorie restriction mimetics (CRM) to support a healthy lifespan.

### 3.3. Relationship Between Diet, Gut Microbiota, and Healthy Aging

Table 2 summarizes the specific diet and microbiota-based interventions evaluated across the seven included studies, along with their reported effects on gut microbiota composition, physical function, cognitive performance, and longevity.

#### 3.3.1. Gut Microbiota Outcomes

Across all seven included studies, Gyriki et al. [19], Xu et al. [20], Luo et al. [21], Li et al. [22], Upadhyay et al. [23], Salazar et al. [24], and Shintani et al. [25] show that dietary interventions consistently demonstrated a strong modulatory effect on the gut microbiota. Mediterranean diet patterns, calorie restriction, vegetarian/vegan diets, and balanced diets have repeatedly been shown to increase the abundance of beneficial short-chain fatty acid (SCFA)-producing bacteria, including *Faecalibacterium*, *Roseburia*, *Bifidobacterium*, *Lactobacillus*, and *Akkermansia muciniphila. At* the same time, these dietary strategies reduced harmful or pro-inflammatory taxa such as *Prevotella*, *Collinsella*, *Ruminococcus torques*, *Escherichia-Shigella*, *Bilophila*, and *Oscillibacter*.

Similarly, microbiota-directed interventions, including probiotics, prebiotics, symbiotics, FMT, and microbial metabolites, produced parallel outcomes. All studies reported an enrichment of beneficial bacterial species, improved microbial diversity, and enhanced SCFA production. Notably, Xu et al. [20] and Luo et al. [21] demonstrated that combining diet with FMT and probiotics produced the most significant microbiota restoration, while Upadhyay et al. [23] highlighted how whole-diet approaches such as the Mediterranean diet and intermittent fasting supported long-term microbial balance. Shintani et al. [25] further showed that calorie-restriction mimetic drugs mimic the microbiota benefits of fasting and the Mediterranean diet.

#### 3.3.2. Physical Outcomes

All included studies reported improvements in physical health associated with diet and microbiota modulation. Gyriki et al. [19], Xu et al. [20], and Luo et al. [21] reported that diets rich in fiber, polyphenols, and plant-based foods can reduce systemic inflammation and improve overall metabolic resilience. Xu et al. [20] showed improved skin health by administering a microbiota drug as well as the probiotic extracts. Across seven studies, increases in SCFA-producers were directly associated with enhanced gut barrier integrity, improved metabolic homeostasis, and reduced inflammatory load, which together promoted healthier physical aging. Even pharmacological mimetics such as those evaluated by Shintani et al. [25] produced similar benefits, decreasing inflammation and increasing the population of beneficial microbiota. According to Upadhyay et al. [23], isoflavones help in healthy aging with the help of probiotics such as (*Lactobacillus helveticus R0052* and *Bifidobacterium longum*), as well as dietary patterns.

#### 3.3.3. Cognitive Outcomes

Cognitive outcomes showed strong associations, as reported by Gyriki et al. [19] and Luo et al. [21], who emphasized that increases in SCFA-producing bacteria reduced neuroinflammation and supported neuroprotection. Xu et al. [20] provided evidence that diet combined with probiotics or FMT could even partially reverse aging-related dementia, likely by restoring healthy hippocampal function. Similarly, Upadhyay et al. [23] highlighted that plant-based diets and fasting enhanced microbial metabolites that support cognitive resilience, while Salazar et al. [24] found that blueberry intake and symbiotic can improve memory and learning through modulation of Sirt1 and FOXO signaling pathways.

#### 3.3.4. Relationship of Diet and Gut Microbiota with Longevity

Across all studies, a clear and consistent relationship emerged between dietary patterns, microbiota modulation, and longevity. The Mediterranean diet was the most frequently associated with increased lifespan, primarily due to its ability to sustain beneficial gut bacteria, reduce inflammation, and maintain metabolic health. Calorie restriction and fasting strategies enhanced longevity by improving microbial diversity and reducing inflammaging. Studies such as Gyriki et al. [19], Luo et al. [21], and Salazar et al. [24] concluded that combining diet with microbiota interventions (probiotics, prebiotics, symbiotics) had the strongest positive effects on healthy aging and lifespan extension. Shintani et al. [25] demonstrated that even mimicking dietary restriction through pharmacological compounds yielded similar longevity benefits by inducing favorable microbiota shifts.

### 3.4. Risk of Bias Assessment

Table 3 illustrates the risk of bias for the seven included reviews assessed by using the SANRA tool [18]. One study by Salazar et al. 2023 [24] was scored as having a low risk of bias, while the remaining six studies [19,20,21,22,23,25] scored medium risk of bias. Most studies consistently failed to provide evidence for item 3, which relates to the description of sources of evidence and justification of selection. Moreover, item 2 was also one of the items that was not completely addressed in the studies. The result of individual scoring for each article is provided in Supplementary Table S3.

## 4. Discussion

This overview aimed to synthesize existing review evidence to examine the roles of gut health and dietary factors in the aging process. There are several interventions, such as the Mediterranean diet, calorie restriction, prebiotic and probiotic supplementation, that foster healthy aging via gut microbiota modulation.

There are several diet norms, such as Western, ketogenic, or high-protein diets; the Mediterranean diet exhibits a greater capacity to augment microbial diversity, elevate the abundance of SCFA-producing bacteria, and diminish pro-inflammatory taxa [12]. These observations align closely with prior studies indicating that Mediterranean and plant-based dietary patterns cultivate a gut environment characterized by an abundance of *Faecalibacterium prausnitzii*, *Roseburia*, and *Akkermansia muciniphila*, all of which are associated with reduced inflammation, enhanced metabolic health, and a postponement of age-related deterioration [13,15,26]. Conversely, diets prevalent in Western societies, which are distinguished by elevated saturated fat levels, refined sugars, and reduced fiber consumption, have been found to be associated with the promotion of dysbiosis and systemic inflammation, thereby accelerating the aging process [27]. Comparative data, consequently, highlights that the quality of the diet, specifically its fiber and polyphenol content, directly influences microbial composition and metabolic output [27,28]. Mechanistically, diets low in fiber provide fewer substrates for saccharolytic fermentation, leading to reduced production of SCFAs such as butyrate, propionate, and acetate, which ordinarily nourish colonocytes and modulate host immune responses [23]. At the same time, high dietary fat and refined protein intake can promote expansion of bile-tolerant and protein-fermenting bacteria, which generate metabolites that may be detrimental to colonic health (e.g., ammonia, phenols, and hydrogen sulfide) and increase inflammatory signaling pathways [28]. High-fat diets have also been shown in animal models to decrease the relative abundance of beneficial bacteria and favor taxa associated with metabolic disturbances and increased inflammatory markers [29]. Moreover, calorie restriction and mimetics, such as metformin and rapamycin, are beneficial supplements that provide metabolic and microbiota benefits linked to conventional Mediterranean diets [21,25]. Although we have found that metformin or any pharmaceutical interventions have been beneficial, there is limited data; therefore, future research should be more focused on such interventions. Normally, microglia, the brain’s resident immune cells, regulate neuron survival and progenitor cells by secreting growth factors. However, during aging, microglia become reactive and imbalanced, causing cognitive dysfunction, changes in brain plasticity, and neurodegeneration [30]. In line with the recent literature, there is increased interest in supplementation along with diet [31]. Probiotics and prebiotics supplementation, along with the MD, have shown an enhanced benefit. In fact, our findings found consistent reports of cognitive improvements, diminished frailty, and more favorable metabolic profiles when dietary modifications were implemented alongside microbiota-targeted therapies [20,21]. Normally, diet strategies prioritize caloric intake or macronutrient distributions, but the Mediterranean approach and microbiota-focused diet comprise a more comprehensive and enduring strategy to promote healthy aging by improving the overall gut microbiota, which has a positive impact on healthy aging. Various studies have stated that interventions such as plant-based, polyphenol-rich, and fiber-dense diets favor and support microbiota, which further impact lifespan and health span [22].

It is important to know that a large portion of the existing literature is regionally concentrated. Most of the studies or interventions that examine the link between diet, microbiota, and aging are based on Mediterranean or Western populations. However, another aspect is based on diets around the world. Evidence from other parts of the world, such as South Asia, Latin America, Japan, China, and Korea, are relatively limited. From a cultural perspective, dietary models from these regions include different combinations of fermented foods, seaweed, legumes, and spices, which may have a positive impact on gut microbiota, but they are not fully studied [32]. For instance, the traditional Japanese diet “washoku” is associated with improved cognition and reduced frailty [33], but it has been observed that Latin American and South Asian populations remain poorly characterized in microbiome research. According to current findings, most of the data is concentrated on the Mediterranean model, with limited understanding of other global dietary patterns and their potential benefits.

However, a specific ideal microbiome profile is unattainable because different factors, such as genetic predispositions, environmental exposures, dietary habits, and pharmaceutical interventions, affect microbial composition [34]. Nevertheless, certain convergent characteristics, such as elevated microbial diversity, a predominance of SCFA-producers, and a reduced presence of pro-inflammatory taxa, consistently characterize healthy aging across diverse populations [34]. As a solution, a personalized nutritional strategy will be useful for customized interventions, and this collective solution will provide both gut and overall health.

### 4.1. Limited Focus on Cognitive Outcomes in Diet–Microbiome Research

Most of the literature is limited mainly to diet–microbiome interactions that focus on metabolic or inflammatory markers, while missing the cognitive health and neurodegenerative outcomes, such as cognitive decline, dementia, and memory loss, which are the main components of aging that remain unexplored in the context. However, the current research has suggested promising links between gut microbial composition and cognitive outcomes, including mild cognitive impairment and Alzheimer’s disease [35].

This lack of evidence is concerning. Cognitive decline not only affects quality of life but also compromises independence, emotional well-being, and long-term care needs. Nonetheless, the current research is in the early stages of establishing the links between gut microbiomes and brain health. Some studies indicate that certain gut bacteria may be linked with cognitive impairment and even Alzheimer’s disease. For example, Tana et al. [35] explored the emerging association between gut microbial patterns and cognitive outcomes. Their findings suggested that SCFAs, produced by gut microbes, may exert neuroprotective effects by maintaining blood–brain barrier integrity, changing microglial activation, and regulating oxidative stress and neuronal metabolism.

Nevertheless, the current research does not provide sufficient in-depth analysis. Still, there remains a clear need to identify specific dietary patterns that consistently support the gut–brain axis, especially in older adults, who are already vulnerable [36]. Therefore, without strong evidence, it is difficult to create practical nutritional plans, preventive programs, or caregiving guidelines that can be helpful to protect cognitive function in the elderly. The scarcity of data highlights a gap in the research. Addressing this gap is essential for future studies, especially as the global population continues to age and the burden of dementia increases. Therefore, including such endpoints in future studies may help to clarify how changes in diet can influence gut microbiota that not only support physical but also cognitive longevity.

In practical terms, European nursing homes and elder care centers follow a broad nutritional guideline for older adults [37,38], which is not typically based on an enforced Mediterranean-style or microbiota-specific diet. We think that strict dietary implementations are often limited due to certain factors such as cost, cultural preferences, and malnutrition risk.

### 4.2. Strengths and Limitations

The data extracted for this review is procured from recent years, proving its time-oriented study (2023–2025). Restricting the search to a 2023–2025 window allowed us to synthesize the most current evidence in a rapidly growing field where, to our knowledge, no prior overview of reviews had been published; nonetheless, a longer window (e.g., five years) could capture additional dietary patterns, populations, and intervention types, and this represents a direction for future overviews of reviews. The studies included are human-based; therefore, their investigation was primarily based on the effect of diet and lifestyle on gut microbiota to support healthy aging. In fact, we observed common themes among the included studies such as an increase in SCFA-producing bacteria, a decrease in pro-inflammatory taxa, along with improved metabolic, immune, and cognitive outcomes with the diverse interventions such as MD, calorie restriction, probiotics, prebiotics, symbiotics, FMT and calorie restriction mimetics. Similarly, the search was restricted to English-language publications for feasibility of systematic screening and data extraction within the resources of this overview, and English was selected because it remains the predominant language of international scientific communication, allowing the inclusion of studies intended for a broad international audience and facilitating comparison across diverse geographical settings. However, this restriction is a language barrier, as we could not include more studies regarding similar topics, specifically about the MD, which is widely studied in Mediterranean countries, where the publications are often in the native language, and it likely contributes to the underrepresentation of non-Western dietary evidence discussed above which gives us a strong concision-based common result and also the potential future research including systematic review and meta-analysis to produce a high level of evidence. Moreover, the study is systematically performed by following PRISMA guidelines, which gives transparency and shows methodological proof.

This overview is also limited by the small number and methodological quality of the included reviews. Of the seven reviews included, only one was assessed as having a low risk of bias, while the remaining six were rated as medium risk of bias. Given this small evidence base and medium risk of bias, the findings presented here should be interpreted as indicative associations rather than firmly established effects. We have also noticed some limitations due to time constraints; many influential and important studies might not have been incorporated in the review, which might have caused the exclusion of certain valuable information. All the studies that are included are narrative reviews, and most do not present general characteristics such as age, sex, etc., which makes it hard to apply the concepts for different individuals. Furthermore, as part of diet interventions, MD stands out as an extensively researched one, leaving other diet contributions in vain, which shows that there is still a scarcity of information on other diets on the human gut influencing longevity. Therefore, combining these limitations of evidence along with the medium risk of bias of included studies, future high-quality systematic reviews and meta-analyses of primary studies are needed to strengthen and validate these findings.

Most nutrition plans emphasize general dietary quality, encouraging higher fruit, vegetable, and whole grain intake without direct microbiome monitoring. As the studies establish the causal link between microbiota composition and aging, future public health strategies can benefit from integrating microbiome parameters into dietary recommendations, healthcare guidelines, and institutional meal planning. To achieve this, there is a need for more studies that test interventions, analyses that weigh costs and benefits, and practical policy plans.

## 5. Conclusions

The present overview highlights the critical role of diet and lifestyle modification in maintaining a healthy gut, which in turn supports healthy longevity. The evidence in our study suggests that MD and caloric restriction diets are fundamental regulators of the aging process, and the targeted supplementations promote beneficial SCFA-producing bacteria that mitigate “inflammaging” and metabolic decline. Maintaining this microbial balance through high-fiber, plant-based interventions appears to support both physical resilience and cognitive health, though evidence on cognitive outcomes in older adults remains particularly limited despite this population’s vulnerability to cognitive decline. These findings should be interpreted with caution, as most of the included reviews were assessed as having a medium risk of bias, and the overall evidence base remains small; as such, the associations reported here should be considered hypothesis-generating rather than conclusive. Future research should prioritize larger, higher-quality reviews and systematic reviews with meta-analysis, address regional gaps beyond Western populations, and explore how microbiome-specific parameters might inform global public health policies and institutional nutrition planning.

## Figures and Tables

**Table 1:** General characteristics of the included studies.

Author and Publication Year	Country	Study Design	Sample Characteristics (Sex and Age)	Aim and Objective of Research
Gyriki et al. 2025 [19]	Greece	Narrative Review	*n* = 2841 (male and female)	To review and examine the relationship between gut microbiota and aging; explore links with age-related diseases; assess potential microbiota-targeting interventions to extend lifespan and improve health outcomes.
Xu et al. 2024 [20]	China	Mini-Review	*n* = 110 (women)	To review the role of gut microbiota as a biomarker of aging; explore microbiota-targeted interventions (FMT, probiotics, diet) to promote healthy aging and longevity.
Luo et al. 2024 [21]	China	Narrative Review	Not specified	To explore mechanisms by which gut microbiota influences healthy longevity; summarize roles of microbiota-derived metabolites (e.g., SCFAs, tryptophan metabolites, bile acids) in enhancing intestinal barrier integrity, reducing inflammaging, optimizing mitochondrial function, regulating nutrient-sensing pathways, and protecting against age-related diseases.
Li et al. 2023 [22]	China	Narrative Review	Not specified	To summarize age-related changes in gut microbiota composition; highlight links with host immunity, nutrition, and signaling pathways; examine the role of dysbiosis in age-related diseases; discuss the potential of gut microbiota in promoting healthy aging and longevity.
Upadhyay et al. 2025 [23]	USA	Narrative Review	*n* = 37	To critically assess age-related changes in gut microbiota and their relationship with age-related diseases; evaluate dietary strategies (fiber, probiotics, prebiotics, polyphenols, Mediterranean diet, fasting); explain mechanisms linking microbiota, inflammation, and chronic diseases; highlight challenges in clinical translation.
Salazar et al. 2023 [24] *	Venezuela	Narrative Review	*n* = 4312; female = 606 (4 studies); male = 286 (1 study)	To explore age-related changes in gut microbiota; examine its role in metabolic, inflammatory, musculoskeletal, and neurological diseases; highlight therapeutic strategies (probiotics, prebiotics, symbiotics, diet, physical activity) to promote healthy aging.
Shintani et al. 2023 [25]	Japan	Narrative Review	*n* = 200	To review evidence on CRM drugs and their effects on gut microbiota; explore whether CRMs can mimic calorie restriction benefits to extend lifespan and promote healthy aging.

Unless otherwise specified, *n* represents the total number of participants reported in the studies, and sex-specific data is reported where available in the original studies; * For Salazar et al. [24], the reported sex-specific figures (female = 606, from 4 studies; male = 286, from 1 study) refer to subsets of participants from individual studies within the review, and are not intended to sum to the review’s overall sample size (*n* = 4312). Abbreviations: CRM, calorie restriction mimetics; FMT, fecal microbiota transplantation; SCFAs, short-chain fatty acids.

**Table 2:** Characteristics of diet and microbiota interventions.

Author and Year	Diet Type	Biota Type	Gut Microbiota Outcomes	Physical Outcomes	Cognitive Outcomes	Main Findings on Longevity
Gyriki et al. 2025 [19]	Mediterranean diet, calorie restriction	Prebiotics, synbiotics	↑ SCFA-producers (*Faecalibacterium*, *Roseburia*, *Bacteroides)*, ↑ *Bifidobacterium*, ↓ harmful taxa	↓ Frailty, ↑ immunity, ↑ metabolic health	↓ Risk of Alzheimer’s disease, ↓ Parkinson’s disease, ↓ cognitive decline	Mediterranean diet ↓ dysbiosis and ↑ healthy aging and longevity
Xu et al. 2024 [20]	Mediterranean diet, calorie restriction, prebiotics	Probiotics, fecal microbiota transplantation	↑ *Lactobacillus*, *Bifidobacterium*, *Akkermansia muciniphila*, SCFA-producers; ↓ *Collinsella*, *R. torques*	↓ Frailty, healthier skin (probiotic extracts), stronger bones, ↑ immunity	Partial reversal of aging-related dementia and cognitive decline via hippocampal restoration	Diet-induced healthy microbiota ↓ inflammaging and ↑ longevity
Luo et al. 2024 [21]	Mediterranean diet, calorie restriction, metformin/rapamycin	Probiotics, fecal microbiota transplantation	↑ *Lactobacillus*, *Bifidobacterium*, *Akkermansia*, SCFA-producers; ↓ pro-inflammatory taxa	↓ Frailty, healthier skin, ↑ immunity, cardiovascular and bone health	↓ Risk of Alzheimer’s and Parkinson’s diseases	Mediterranean diet and microbiota modulation ↑ anti-aging effects and longevity
Li et al. 2023 [22]	Balanced diet, prebiotics	Probiotics, fecal microbiota transplantation, microbial metabolites	↑ *Bifidobacterium*, *Lactobacillus*, *Akkermansia*, SCFA-producers; ↓ pathogenic taxa	↑ Immunity, healthier skin, metabolic balance	↓ Frailty and neuroprotection	Balanced diet and microbiota ↓ inflammaging and ↑ longevity and metabolic stability
Upadhyay et al. 2025 [23]	Mediterranean diet, intermittent fasting, vegetarian/vegan diets	Probiotics	↑ *Bifidobacterium*, *Lactobacillus*, *Faecalibacterium*, *Roseburia*, *Akkermansia muciniphila*	Protection against cardiovascular disease, diabetes, and AMD	Protection against neurodegeneration and age-related cognitive decline	Microbiota-focused diets ↓ dysbiosis and support healthy aging and longevity
Salazar et al. 2023 [24]	Mediterranean diet, prebiotics, symbiotics, healthy diet, blueberry intake	Probiotics	↑ *Akkermansia*, *Lactobacillus*, *Bifidobacterium*, ↑ SCFAs; ↓ *Prevotella*	↑ Muscle function, bone health (some studies), ↓ inflammation	↑ Memory and learning (via SIRT1, FOXO1/3 modulation)	Mediterranean diet with probiotics/prebiotics ↓ inflammaging and increases longevity
Shintani et al. 2023 [25]	Calorie-restriction mimetic drugs and compounds	—	↑ *Akkermansia*, *Bifidobacterium*, *Lactobacillus*, *Faecalibacterium;* ↓ *Escherichia-Shigella*, *Bilophila*, *Oscillibacter*	↑ Metabolism, ↓ inflammation	↓ Risk of Alzheimer’s and Parkinson’s diseases	Calorie restriction mimetics ↑ lifespan extension and healthy aging

Abbreviations: AMD, age-related macular degeneration; SCFA, short-chain fatty acid-producing bacteria. Arrows denote an increase (↑) or decrease (↓).

**Table 3:** Risk of bias assessment (SANRA tool).

Author and Year	Study Design	Risk of Bias
Gyriki et al. 2025 [19]	Narrative Review	Medium
Luo et al. 2024 [21]	Narrative Review	Medium
Li et al. 2023 [22]	Narrative Review	Medium
Upadhyay et al. 2025 [23]	Narrative Review	Medium
Salazar et al. 2023 [24]	Narrative Review	Low
Shintani et al. 2023 [25]	Narrative Review	Medium
Xu et al. 2024 [20]	Mini-Review	Medium

Abbreviation: SANRA, Scale for the Assessment of Narrative Review Articles.

## Data Availability

No new data were created or analyzed in this study. Data sharing is not applicable to this article.

## Supplementary Materials

The following supporting information can be downloaded at: https://www.mdpi.com/article/10.3390/geriatrics11040107/s1, Table S1: PRISMA 2020 Checklist—Geriatrics (MDPI); Table S2: Search Strategy with keywords; Table S3: SANRA risk of bias assessment of included reviews.

## Abbreviations

The following abbreviations are used in this manuscript:

AMD	Age-related Macular Degeneration
CRM	Calorie Restriction Mimetics
FMT	Fecal Microbiota Transplantation
FOXO	Forkhead Box O
MD	Mediterranean Diet
OECD	Organization for Economic Co-operation and Development
PICOS	Population, Intervention, Comparison, Outcome, Study Design
PRISMA	Preferred Reporting Items for Systematic Reviews and Meta-Analyses
SANRA	Scale for the Assessment of Narrative Review Articles
SCFA	Short-Chain Fatty Acid
SCFAs	Short-Chain Fatty Acids
SIRT1	Sirtuin 1
TMAO	Trimethylamine-N-Oxide
MeSH	Medical Subject Headings
